# The Effect of Immigration on Adverse Perinatal Outcomes: Analysis of Experiences at a Turkish Tertiary Hospital

**DOI:** 10.1155/2019/2326797

**Published:** 2019-11-05

**Authors:** Ilknur Col Madendag, Mefkure Eraslan Sahin, Yusuf Madendag, Erdem Sahin, Mustafa Bertan Demir, Fatma Ozdemir, Gokhan Acmaz, Iptisam Ipek Muderris

**Affiliations:** ^1^Department of Obstetrics and Gynecology, Kayseri City Hospital, Kayseri, Turkey; ^2^Department of Obstetrics and Gynecology, Erciyes University Medicine Faculty, Kayseri, Turkey

## Abstract

**Introduction:**

In literature, it is well documented that migration is associated with adverse perinatal outcomes in many countries over the world. But in Turkey, health care providers and obstetricians had to face the effects of migration for the first time after civil war in Syria. Hence, this situation motivated us to conduct the current research in Turkey. Also we aimed to evaluate the effect of immigration on adverse perinatal outcomes, comparing the obstetric results of a native population and an immigrant population, and focusing on relevant indicators of perinatal health.

**Methods:**

Information from the hospital database of pregnant women who had vaginal or cesarean delivery was evaluated. The patients were divided into two groups, native women and immigrant women, according to their ethnic origin. Adverse perinatal outcomes were compared between groups using multivariate regression models. Adjusted odds ratio (aOR) and 95% confidence interval (CI) were calculated.

**Results:**

A total of 6311 patients were evaluated, of which 4271 were classified as native and 2040 were classified as immigrants. Mean hemoglobin level before delivery was significantly lower in the immigrant group. Preterm delivery (aOR: 1.41; 95% CI: 1.19–1.65), stillbirth (aOR: 1.88; 95% CI: 1.09–3.23), red blood cell transfusion requirement (aOR: 3.12; 95% CI: 2.02–3.98), unplanned birth rates before hospital arrival (aOR: 2.25; 95% CI: 1.53–3.31), and postpartum infection rates (aOR:2.12; 95% CI: 1.48–3.08) were significantly increased in the immigrant group compared with native group, even considering adjustment for potential confounders.

**Conclusion:**

The immigration may be an important and independent risk factor for some adverse maternal and neonatal outcomes.

## 1. Introduction

Care provided during pregnancy and labor is an important consideration in every nation. As a result of migration, different cultures may meet at child birth which is a very sensitive moment in life. In addition to personal encounters, pregnant refugee women and their families from different parts of the world come in contact with doctors, midwives, and maternal health care staff in general and are confronted by what may be unfamiliar institutional cultures and practices [1]. As increasingly ethnically different immigrant women resettle in developed countries, several factors should be considered when caring for these individuals during pregnancy and labor [1].

Migration is increasing due to a variety of reasons including socioeconomic concerns, civil war, and drought. Due to the ongoing civil war in Syria, there are many immigrants in Turkey and this number is increasing. Currently, considering the data in September 2019, it is possible to say that there are 3 million 666 thousand migrants. Of them, 1 million 679 thousand were women and 405 thousand immigrant babies were born in the last 10 years in Turkey. Reproductive health, pregnancy, and the postpartum period may be affected as a result of sociocultural differences. Immigrants may come from different economies and environments, with different cleaning and eating habits, diverse bacterial flora, and intermittent access to health care services [2]. The rich-poor gap has been largely studied and reported as one of the causes of adverse postnatal outcomes [3], but in Turkey, similar to many countries, perinatal follow-up, hospitalization, surgical procedures, and necessary medication are provided free of charge for the immigrant population as required by the health policy. Thus, it can be easily said that economic problems are not the factor for adverse perinatal outcomes in immigrant population in Turkey. As a matter of fact, in many research studies conducted with immigrant women, it has been reported that adverse pregnancy outcomes such as stillbirth, perinatal mortality, rate of cesarean delivery, preterm delivery, and low birth weight (LBW) are higher than in the native population [1, 4–7]. In literature, it is well documented that migration is associated with adverse perinatal outcomes in many countries over the world. But in Turkey, health care providers and obstetricians had to face the effects of migration for the first time after civil war in Syria. Hence, this situation motivated us to conduct the current research in Turkey. Also we aimed to evaluate the effect of immigration on adverse perinatal outcomes, comparing the obstetric results of a native population and an immigrant population, and focusing on relevant indicators of perinatal health.

## 2. Materials and Methods

This study was performed retrospectively in Kayseri City Hospital Obstetrics and Gynecology Clinic. All steps of the study were performed in accordance with the Declaration of Helsinki and with the approval of the 2018/359 Erciyes University Medical Faculty Clinical Research Ethics Committee.

From May 2018 to February 2019, information from the hospital database for all pregnant women (*n*: 7115) who delivered with spontaneous vaginal births or cesarean sections was evaluated. Patients with preeclampsia, eclampsia, gestational hypertension, type 1 or 2 diabetes, gestational diabetes, intrahepatic cholestasis of pregnancy, placental invasion anomalies, and known congenital or chromosomal fetal anomalies were excluded from the study (*n*: 804/7115). The patients were divided into two groups according to their ethnic origin: native Turkish women and immigrants.

The gestational weeks of the patients were determined according to their last menstrual period if known or calculated with first trimester ultrasonography if unsure. Relevant adverse perinatal outcome indicators were as follows: preterm rates (gestational age <37 weeks), LBW <2.500 g, and very LBW <1.500 g rates, mode of delivery and condition of the genitals after vaginal delivery, 5-minute Apgar scores <7, length of neonatal intensive care unit (NICU) stay, and stillbirth rates. Postpartum infection was defined as the presence of any postpartum endometritis, postcesarean wound infection, or postepisiotomy wound infection. Postpartum hemorrhage value (g/dL) was calculated as the difference between pre- and postnatal hemoglobin values for all patients.

## 3. Statistical Analysis

Statistical analysis was performed with Minitab 16 (Minitab Inc., State College, PA, USA). Categorical data are presented as count and proportions. Groups were compared using Pearson's chi-square test or Fisher's exact test. Continuous data are presented as mean ± standard deviation (SD) or median (25–75 percentiles), and groups were compared using a *t*-test or Mann–Whitney *U* test. A two-sided *p* value of 0.05 was considered statistically significant.

Multivariable logistic or linear regression analysis was used to compare between groups for maternal and neonatal outcomes. For these comparisons, age, BMI, and parity were considered as potential confounder. There was a significant difference for only age between groups. Hence, only the age-adjusted odds ratio was computed using logistic regression with 95% confidence intervals (CI). Age-adjusted odds ratio was calculated only for categorical data, and not continuous data. Previous cesarean rate was also considered as a potential confounder to compare between groups for cesarean section. Hence, age and previous cesarean adjusted odds ratio was calculated for cesarean section.

## 4. Results

A total of 6311 patients were evaluated, of which 4271 were classified as native patients and 2040 as immigrants. A total of 92% of the immigrant group consisted of Syrian patients and the remaining women came from Iraq (3.5%), Afghanistan (1.5%), China (1.5%), Azerbaijan (%1), and Algeria (0.5%).

Maternal demographic characteristics are shown in Table 1. In comparison, the mean maternal age was lower in the immigrant population group compared to the native group (23.7 ± 5.7 versus 26.9 ± 5.9 years, *p* < 0.001). The birth rate under 20 years of age was higher in immigrant patients, and the rate of birth above 35 years was higher in native patients (*p* < 0.001, *p* < 0.001, respectively). Body mass index (BMI), nulliparity, gravidity, and multiple pregnancy rates were found to be similar in both groups (*p*=0.498, *p*=0.409, *p*=0.457, and *p*=0.702, respectively). Previous cesarean section rates were statistically higher in the native group compared to the immigrant group (*p* < 0.001).

Adverse maternal outcomes were compared and illustrated in Table 2. The cesarean section rate was higher in the native group compared to the immigrant group (36.8% versus 21.1%, *p* < 0.001, adjusted for age and previous cesarean rate OR = 0.64, 95% CI: 0.53–0.78). The 3rd/4th degree tear rates, hospital stay, and mean postpartum hemorrhage values were similar in both groups (*p*=0.628, *p*=0.205, and *p*=0.820, respectively). Mean hemoglobin level before delivery was significantly lower in the immigrant group (*p* < 0.001), and red blood cell transfusion requirement (0.6% versus 2%, *p* < 0.001, adjusted for age OR = 3.12, 95% CI: 2.02–3.98), unplanned birth rates before hospital arrival (1.2% versus 2.6%, *p* < 0.001, adjusted for age OR = 2.25, 95% CI: 1.53–3.31), and postpartum infection rates (1.3% versus 2.5%, *p* < 0.001, adjusted for age OR = 2.12, 95% CI: 1.48–3.08) were significantly increased in the immigrant group compared to the native group.

Adverse neonatal outcomes are shown in Table 3. Prematurity was significantly increased in the immigrant group compared to the native group (9.8% versus 13.2%, *p* < 0.001, adjusted for age OR = 1.41, 95% CI: 1.19–1.65). Although LBW (<2500 g) and very LBW (<1500 g) rates were similar, mean birth weight was lower in the immigrant group compared to the native group (*p*=0.048). The 5-minute Apgar score <7 and admission to the NICU rates were similar in both groups (*p*=0.742 and *p*=0.275, respectively). The stillbirth rate was higher in the immigrant group compared to the native group (0.7% versus 1.2%, *p*=0.028, adjusted for age OR = 1.88, 95% CI: 1.09–3.23).

## 5. Discussion

In the present study, we aimed to evaluate the effect of immigration on adverse perinatal outcomes in a reference tertiary hospital in Turkey. We found immigration to be an important and independent risk factor for some adverse maternal and neonatal outcomes such as the need for blood transfusion, unplanned birth before hospital arrival, postpartum infection rate, preterm birth (<37 week), and stillbirth, even considering adjustment for age.

When we looked at maternal demographic characteristics, we found that maternal age and previous cesarean section rates were higher in native patients and the birth rate under 20 years of age was higher in immigrant patients. It is possible to associate the higher maternal age in native patients with the education levels of these women, the time they spent on their education, and whether they had a profession. The fact that prior cesarean rates were lower in the immigrant population may be a result of negative views towards this surgery in various cultures of the refugees, prompting resistance to the procedure. We did not find any significant differences in the other demographic characteristics compared between the two groups.

When maternal outcomes were evaluated, we found that native patients had higher cesarean section rates compared to immigrant patients. Like this prior cesarean delivery rate was higher in the native population than in the immigrant group. There are several studies evaluating cesarean rates in the immigrant population in the literature, some studies have found a high rate of cesarean section in the immigrant population [8], while in another study the rate of cesarean sections has been reported as higher in the native population [7]. This situation may be related to ethnicity, socioeconomic status, and sociocultural factors [9–11]. We speculated that almost all of the immigrant group consisted of Syrian patients who belong to Arabian ethnic group and almost all of the native group consisted of Turks patients who belong to Caucasian ethnic group. Although both societies are from border countries, they have different anatomic features such as body height, muscle, and fat mass. These anatomic variations may be the reason of the difference of cesarean rate between the groups.

Additionally, we found that the red blood cell transfusion requirement, unplanned birth rates before hospital arrival, and postpartum infection rates were significantly increased in the immigrant group compared to the native group. When postpartum hemorrhage rates are similar, the fact that the transfusion rate is higher in the immigrant population can be related to lower predelivery maternal hemoglobin levels. A high rate of postpartum infection rates in the immigrant group can be associated with low socioeconomic status, environmental factors, malnutrition, and higher rates of unplanned birth before hospital arrival [12].

In the current study, we found that the risk for preterm delivery was 41% higher in the immigrant group compared to the native group. Since we excluded high-risk pregnancies associated with prematurity, such as preeclampsia, diabetes, and pregnancy cholestasis, we can theorize that immigration is an important and independent factor for prematurity, even considering adjustment for age. Although there is a slight decrease in the mean birth weight of the immigrant group which is not clinically significant, unlike other studies [13], LBW (<2500 g) and very LBW (<1500 g) rates were similar in both groups. One of the parameters of the adverse neonatal outcome is undoubtedly stillbirth. Similar to other studies [14], we found that the stillbirth rate was significantly increased in the immigrant group 1.88-fold. We did not find any statistical difference between the two groups for other important parameters including the 5-minute Apgar score <7 or admission to the NICU.

The present study contains both limitations and strengths. Important limitations include the fact that the study was retrospective in design and 92% of the study population consisted of Syrian migrants; therefore, a subgroup analysis of other ethnicities could not be performed. Another limitation was that the study was done in a single center and there were not enough data about socioeconomic status, or education level, or number of antenatal visits in the hospital databank. On the other hand, the large number of patients in the groups evaluated and the exclusion of high-risk pregnancies are strengths of the study.

## 6. Conclusion

The immigration may be an important and independent risk factor for some adverse maternal and neonatal outcomes such as the need for blood transfusion, unplanned birth before hospital arrival, postpartum infection rate, preterm birth (<37 weeks), and stillbirth. Further studies with multicenter are needed.

## Figures and Tables

**Table 1:** Comparison of maternal demographic characteristics.

	Natives (*n* = 4271)	Immigrants (*n* = 2040)	*p* value
Age (years)	26 (22–31), 12–52	22 (20–27), 13–45	<0.001
Age <20	377 (8.8%)	507 (24.9%)	<0.001^*∗*^
Age >35	424 (9.9%)	95 (4.7%)	<0.001^*∗*^
BMI (kg/m^2^)	25.3 ± 2.1	25.9 ± 1.9	0.498
Nulliparity	1072 (25.1%)	491 (%24.1)	0.409^*∗*^
Gravidity	2.96 ± 1.51	2.53 ± 1.54	0.457
Multiple pregnancy	51 (1.2%)	22 (1.1%)	0.702^*∗*^
Prior cesarean section	1062 (25.2%)	268 (13.3%)	<0.001^*∗*^

BMI: body mass index. ^*∗*^Chi-square. Values are expressed as *n* (%), or median (25–75%), or mean ± SD.

**Table 2:** Comparison of adverse maternal outcomes.

	Natives (*n* = 4271)	Immigrants (*n* = 2040)	*p* value	Adjusted odds ratio (95% CI)
Cesarean section	1573 (36.8%)	430 (21.1%)	<0.001^*∗*^	0.64 (0.53–0.78)^b^
3rd/4th degree tears (vaginal births)	33/2560 (1.3%)	19/1570 (1.2%)	0.628^*∗*^	0.96 (0.88–1.36)^c^
Maternal length of hospital stay (days)	1.58 ± 0.72	1.42 ± 1.12	0.205	
Postpartum hemorrhage value^a^ (g/dL)	0.96 ± 0.72	1.01 ± 0.78	0.820	
Mean hemoglobin level before delivery (g/dl)	10.5 ± 2.08	9.1 ± 1.78	<0.001	
The need for blood transfusion	26 (0.6%)	41 (2%)	<0.001^*∗*^	3.12 (2.02–3.98)^c^
Unplanned birth before hospital arrival	51 (1.2%)	54 (2.6%)	<0.001^*∗*^	2.25 (1.53–3.31)^c^
Postpartum infection rate	32 (1.3%)	51 (2.5%)	<0.001^*∗*^	2.12 (1.48–3.08)^c^

CI: confidence interval. Values are expressed as *n* (%) or mean ± SD. ^*∗*^Chi-square. ^a^Postpartum hemorrhage value was calculated as the difference between pre- and postnatal hemoglobin values for all patients. ^b^Odds ratio adjusted for age and previous cesarean. ^c^Odds ratio adjusted for age.

**Table 3:** Comparison of adverse neonatal outcomes.

	Natives (*n* = 4271)	Immigrants (*n* = 2040)	*p* value	Adjusted odds ratio^a^ (95% CI)
Preterm birth (<37 weeks)	418 (9.8%)	270 (13.2%)	<0.001^*∗*^	1.41 (1.19–1.65)
Fetal gender (male)	2203 (51.6%)	1046 (51.3%)	0.820^*∗*^	
Birth weight (g)	3210 (2920–3520)	3155 (2885–3430)	0.048	
LBW <2500 g	324 (7.6%)	151 (7.4%)	0.795^*∗*^	0.96 (0.80–1.22)
Very LBW <1500 g	50 (1.2%)	24 (1.2%)	0.959^*∗*^	1.06 (0.92–1.12)
5-minute Apgar score<7	85 (2%)	47 (2.3%)	0.742^*∗*^	1.15 (0.98–1.80)
NICU (days)	0.60 ± 2.40	0.76 ± 3.14	0.275	
Stillbirth	28 (0.7%)	25 (1.2%)	0.028^*∗*^	1.88 (1.09–3.23)

CI: confidence interval; LBW: low birth weight; NICU: neonatal intensive care unit. Values are expressed as *n* (%), or median (25–75%), or mean ± SD. ^*∗*^Chi-square. ^a^Odds ratio adjusted for age.

## Data Availability

Data used to support the findings of this study are available from the corresponding author upon request. All data belong to Kayseri City Hospital.
